# Interspecific Hybridization in Pilot Whales and Asymmetric Genetic Introgression in Northern *Globicephala melas* under the Scenario of Global Warming

**DOI:** 10.1371/journal.pone.0160080

**Published:** 2016-08-10

**Authors:** Laura Miralles, Marc Oremus, Mónica A. Silva, Serge Planes, Eva Garcia-Vazquez

**Affiliations:** 1 Department of Functional Biology, University of Oviedo, 33006, Oviedo, Spain; 2 16 rue Henri Niautou, 98800, Noumea, New Caledonia; 3 MARE–Marine and Environmental Sciences Centre and Centre of IMAR- Institute of Marine Research, University of the Azores, 9901–862, Horta, Portugal; 4 Biology Department, Woods Hole Oceanographic Institution, Woods Hole, MA, 02543, United States of America; 5 Laboratoire d’Excellence “CORAIL”, Centre de Recherche Insulaire et Observatoire de l'Environnement (CRIOBE), USR 3278 CNRS-EPHE-UPVD, BP 1013 Papetoai, 98729, Moorea, Polynésie Française; Virginia Tech Virginia, UNITED STATES

## Abstract

Pilot whales are two cetacean species (*Globicephala melas* and *G*. *macrorhynchus*) whose distributions are correlated with water temperature and partially overlap in some areas like the North Atlantic Ocean. In the context of global warming, distribution range shifts are expected to occur in species affected by temperature. Consequently, a northward displacement of the tropical pilot whale *G*. *macrorynchus* is expected, eventually leading to increased secondary contact areas and opportunities for interspecific hybridization. Here, we describe genetic evidences of recurrent hybridization between pilot whales in northeast Atlantic Ocean. Based on mitochondrial DNA sequences and microsatellite loci, asymmetric introgression of *G*. *macrorhynchus* genes into *G*. *melas* was observed. For the latter species, a significant correlation was found between historical population growth rate estimates and paleotemperature oscillations. Introgressive hybridization, current temperature increases and lower genetic variation in *G*. *melas* suggest that this species could be at risk in its northern range. Under increasing environmental and human-mediated stressors in the North Atlantic Ocean, it seems recommendable to develop a conservation program for *G*. *melas*.

## Introduction

Global environmental changes, including temperature increase, glacial ice melting and sea level rising, are intense in the northern latitudes where they are reshaping the fragile local ecosystems with potentially devastating consequences for vulnerable species [1]. Amongst other consequences of global change, a general increase of interspecific hybridization is predicted in Polar and subpolar zones due to the loss of temperature barriers [2] and the arrival of new species displaced from warmer zones. Although introgressive hybridization occurs rarely in nature [3], it is more frequent in fast-changing ecosystems. Species may alter their mate choice under altered or adverse conditions, subsequently acquiring new genetic variation [4]. Interspecific hybridization is especially scarce in mammals [5]; the exception is cetaceans for which many hybrids occur e.g. [6, 7], not only between species within a genus but also between different genera [8]. Cetacean species experiencing distribution shifts could be good models to test Kelly et al. [2] prediction of an increase of hybridization caused by global warming.

Long-finned (*Globicephala melas*) and short-finned (*Globicephala macrorhynchus*) pilot whales are two sympatric cetaceans of Delphinidae, a family under recent speciation [9, 10] and a controversial number of species [11, 12]. Based on osteological data, Van Bree [13] demonstrated that pilot whales are two clearly distinct species, which was later supported by molecular phylogenetic studies [9, 14, 15]. Predicted impacts of global climate change on the marine environment may induce changes in pilot whales species range, abundance and/or migration patterns [16, 17]. The distribution of *G*. *melas* is correlated with sea surface temperature [18–21]. Moreover, at the northern limits of its range, *G*. *melas* abundance is also correlated with the North Atlantic climate oscillation [21–23]. *G*. *macrorhynchus* also seems to be affected by climate variations [24, 25]. On the other hand, pilot whale distribution is primarily associated with prey abundance (e.g. [20, 24, 26, 27]), which in turn is also influenced by temperature [21, 25, 28].

The genetic identification of one post-F1 hybrid pilot whale in the Northeast Atlantic Ocean [29] demonstrated that *G*. *melas* and *G*. *macrorhynchus* are able to hybridize successfully. Twenty years before this finding, a Faeroese study [30] reported clines of external morphological traits of pilot whales, instead of clear diagnostic features: 2.7% of *G*. *melas* off the Faeroe Islands exhibited morphometric traits typical of *G*. *macrorhynchus*. This scenario makes the Northeastern Atlantic pilot whales a good model for investigating the association between climate change and interspecific hybridization in cold-temperate latitudes. We analysed information from different disciplines to infer the effect of global climate change on the sympatric distribution of *G*. *melas* and *G*. *macrorhynchus* at the northernmost limit of their range. Microsatellite markers with species-specific alleles and coalescent demographic reconstructions from DNA sequences were combined with oceanographic data and paleo-temperature reconstructions, for tracing the evolutionary history of these species. The departure hypothesis was that climate warming alterations could promote population genetic alterations (like population size reduction, interspecific hybridization, etc.) in pilot whales from the colder waters of North Atlantic regions.

## Material and Methods

### Oceanographic and temperature reconstructions

GISS temperature analysis [31, 32] was used to calculate trends in annual temperature change from 1914 to 2014. We also estimated changes in sea surface temperature (SST) per month during the 1980–2015 period, and the seasonal mean SST anomalies per year at all latitudes from 1988 to 2013 using the database SST-Hadley/Reynols. All estimations were calibrated with a base period from 1951 to 1980 [31].

Climate reconstruction focused on the known reproduction seasons of North Atlantic pilot whales: May-October for *G*. *melas* [33, 34] and July-August for *G*. *macrorhynchus* [35].

For paleoclimatic reconstructions, temperatures were obtained from Marcott et al. [36]. We used the core MD95-2015 with the proxy UK´37, calibrated with Müller et al. [37] for marine ages of North Atlantic annual temperatures [38].

### Pilot whale samples

A total of 151 samples of the two pilot whale species were collected from four different locations between 1997 and 2012 (Faeroe Islands, coasts of the Iberian Peninsula, Azores Islands and Canary Islands). A vicariant population outgroup of *G*. *macrorhynchus* was obtained from French Polynesia, Pacific Ocean. Tissue samples were taken from stranded animals, biopsies and museum collections. An attempt was made to avoid sampling more than one member from the same family group. No animal was injured or killed for this study. All protocols and analyses were approved by the Committee of Ethics of the University of Oviedo. We obtained the CITES permit (ESBI00001/12I) and all the permissions from the Faroese Museum of Natural History to bring the Faroese samples to Spain, as well as French Polynesian samples (CITES permit 13NZ000012; original permit number FR-02-987-0083-E).

### Genetic analysis

DNA was extracted employing a Chelex-based protocol [39]. The mitochondrial control region (D-loop) was amplified following Oremus et al. [15]. Sequences were edited and aligned using ClustalW [40] from the BioEdit Sequence Alignment Editor [41]. NCBI-BLAST [42] online software was employed for species identification. The number of haplotypes, haplotypic and nucleotidic diversities were calculated with DNAsp v5 [43]. A median-joining [44] haplotype network was constructed to visualize the relationships among the different mitochondrial haplotypes, and their frequencies, in the sampled populations with the program Network 4.5.1.6 [45]. Network software reconstructs all possible, shortest, least complex phylogenetic trees (maximum parsimony) from a given dataset using different algorithms.

Eight microsatellite loci (EV37MN; EV94MN; 199/200; 415/416, 417/418, 409/470; 468/469 and 464/465) were amplified as in Fullard et al. [20]. A multi-tube method [46] was employed to validate the allele scores. Each microsatellite locus was individually amplified three times in three different thermal cycler machines (Applied Biosystems 2720 Thermal Cycler). Microsatellites were genotyped employing GeneMapper® Software Version 4.0 (Applied Biosystems). Scoring errors, large allele dropout and null alleles were tested employing the program MICROCHECKER [47].

Samples with positive amplification for all mitochondrial and microsatellite loci were sexed by amplifying the Y-linked SRY gene [48], typical of males. PCR was run with positive and negative controls to avoid possible errors. PCR products were run and visualized in 2% agarose gel.

### Population genetics

Conformity with Hardy-Weinberg equilibrium was calculated using the exact probability test with GENEPOP software [49] and Bonferroni corrections. Microsatellite variation (number of alleles per locus, allelic richness and observed and expected heterozygosity) was calculated with the programs GENETIX Version 4.03 [50] and FSTAT Version 2.9.3.2 [51]. Genetic divergence between populations was estimated using population pairwise F_ST_ values obtained with Arlequin version 3.0 [52]. To detect recent bottleneck events we employed the software BOTTLENECK version 1.2.02 [53] with default settings.

### Evolutionary and demographic inferences

Population divergence time estimations were computed under a Bayesian Markov Chain Monte Carlo (MCMC) framework using BEAST version 1.6.1 [54]. Following a burn-in of 5 million cycles, rates were sampled once every 1 000 cycles from 50 million MCMC steps for an Extended Bayesian Skyline tree, with a stepwise model for mitochondrial DNA and an evolution rate of 1.5% per million years [55–57]. Bayesian intraspecific phylogenies are based on coalescent theory [58] and allow the inference of past population dynamics and parameters from contemporary gene sequences. The best evolution model and its priors (kappa, gamma-shape, proportion of invariant sites, etc.) were defined by jModeltest software version 0.11 [59] using the Akaike information criterion [60]. Tracer version 1.5 [54] was used to check chains had converged to a stationary distribution.

### Interspecific genetic relationships

The software STRUCTURE v.2.3.1 [61] was employed for estimating the membership of each individual to each species (K = 2; two expected genetic units) under the “Admixture model” which assumes that individuals may have mixed ancestry. Settings were a burn-in period of 70000 steps followed by 700000 MCMC iterations and seven runs. Since there is no clear consensus about the proportion of membership that indicates introgression [62], for conservative interpretation we have considered >25% as a threshold for significant introgression [29]. Confidence intervals of 95% were calculated for all membership values. The software NewHybrids [62] was run for identifying individuals of each pure species, hybrids of first and second generation and backcrosses. An initial run was implemented for only pure species individuals with >0.980 membership detected from STRUCTURE, in order to assess the discrimination power of our dataset in NewHybrids. A second run was done for 10 hypothetical F1 hybrids and pure species. Then, the MCMC was run for 500000 iterations after a burn-in period of 50000 iterations for our original data set.

Gene flow between species in the North Atlantic Ocean was estimated with MIGRATE 3.0 [63] from Ө = xNeμ and M = m/μ (x, inheritance parameter; Ne, effective size; m, immigration rate; μ, mutation rate) in each species based on coalescent theory [64] and relaxing the original assumption of Wright [65]. If Ө and M are multiplied together, the number of immigrants per generation can be calculated as gene flow. We used this formula, employing the inheritance parameter x (x = 4 for nuclear DNA [here microsatellite loci] and 1 for mtDNA sequences), to calculate the effective number of immigrants per generation for nuclear and mitochondrial DNA separately. To be sure that results do not reflect spurious local likelihood peaks, three independent runs were performed with a Bayesian approximation to ensure final chains were estimating the same value of Theta (Ө), burn-in of 500000, fifteen long chains (50000 recorded steps with increment of 100) and five replicates. Gene flow between species was also calculated from microsatellite data using the private alleles method implemented in GENEPOP [49].

## Results

### Temperature reconstructions

**Fig 1:**
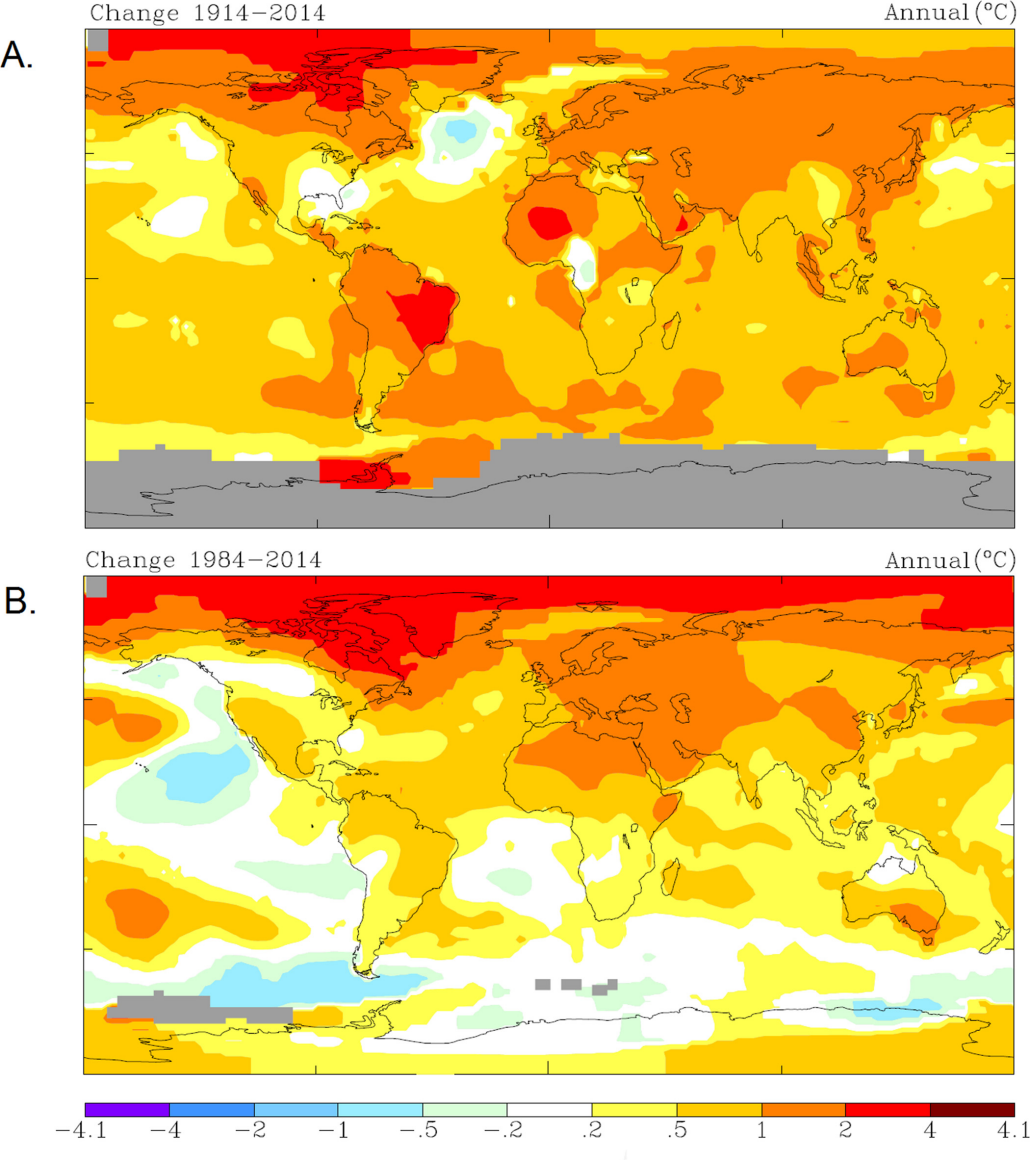
Global temperature changes. A. GISS surface temperature analysis [32] taken from NASA by using annual temperature change trends from 1914 to 2014 and calibrated with a base period from 1951 to 1980. B. GISS surface temperature analysis [32] taken from NASA by using annual temperature change trends from 1984 to 2014 and calibrated with a base period from 1951 to 1980. Gray color means data missing.

**Fig 2:**
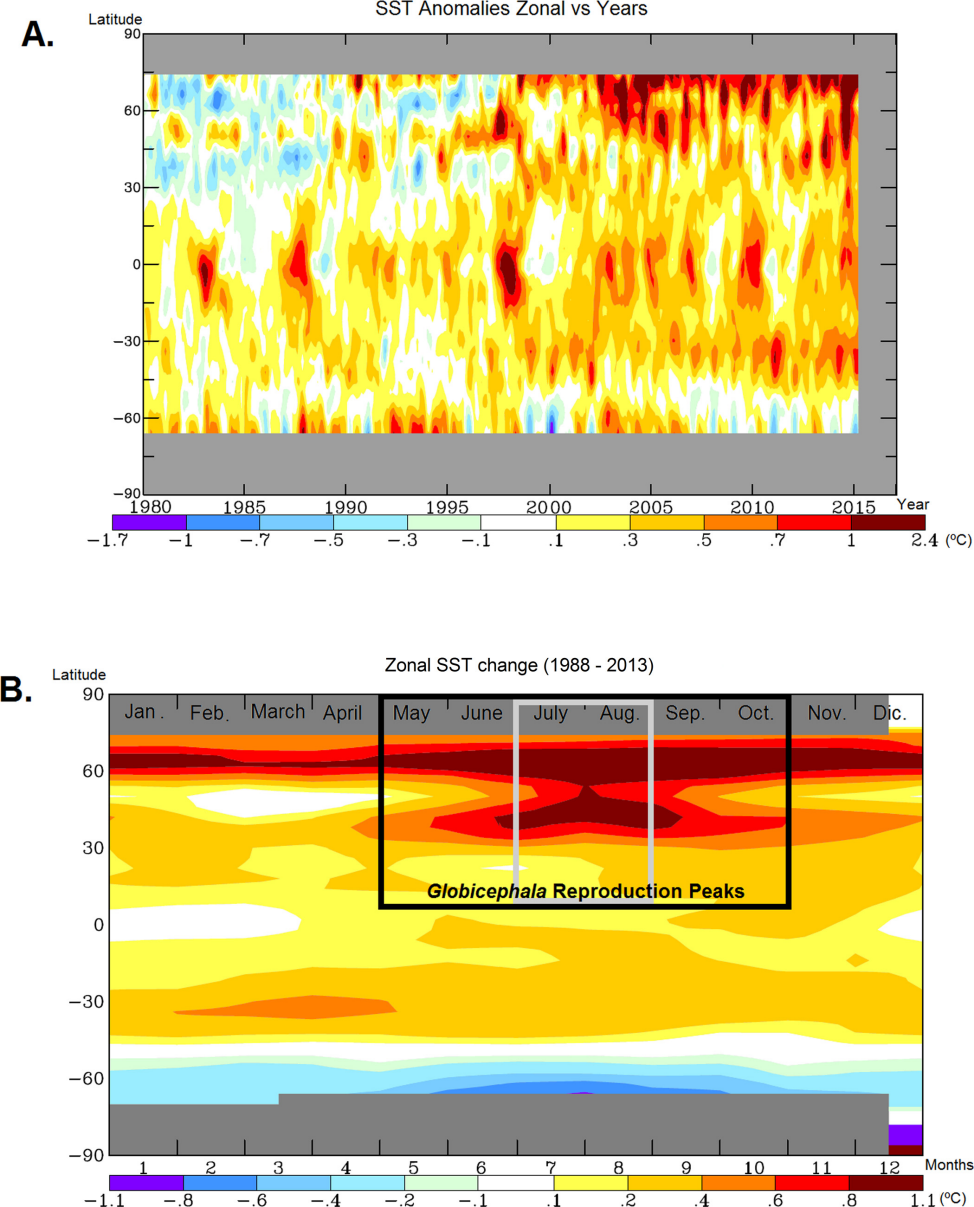
Sea surface temperature (SST). **A.** SST anomalies represented by zonal means versus years from 1980 to 2015. Data base: Sea Surface Temperatures (°C)—Hadley/Reynolds. Base period: 1951–1980. GISS surface temperature analysis [32]. B. SST change represented by zonal means from 1988 to 2013 versus Months. Data base: Sea Surface Temperatures (°C)—Hadley/Reynolds. Base period: 1951–1980. GISS surface temperature analysis [32]. Reproduction seasons are represented with a black square for *Globicephala melas* and a grey square for *G*. *macrorhynchus*.

Temperature increased globally in the last hundred years (Fig 1A), especially in the Northeast Atlantic Ocean for the last decades (Fig 1B). Air temperature increased there two Celsius degrees between 1984 and 2014. Focusing on sea surface temperature (SST) anomalies, water temperature increased up to 2.4°C in the last ten years (Fig 2A). The increase in SST was greater in the northern latitudes (Fig 2A and 2B). There, the SST warming was more intense during summer months (Fig 2B), coincident with the reproduction season of pilot whales. Furthermore, at this time of the year the SST warming affected a wider area from 30°N to the northernmost latitude. This temperature increase may favour the northward expansion of *G*. *macrorhynchus*, thereby promoting the overlap in distribution between the two species during the reproduction season. This coincidence provides the climate context for further results on population genetics of these species.

### Population genetics

A total of 120 DNA samples provided positive PCR amplification for all mitochondrial and nuclear genetic markers considered. These samples were genetically sexed. More males than females were analyzed for the two species (Table 1): 32 males versus 31 females for *G*. *macrorhynchus* and 50 males versus 7 females for *G*. *melas*.

**Table 1:** Genetic diversity for each species and location.

Species			Sex		D-LOOP			Microsatellite loci			
	Localities	N	(M: F)	Nh	Hd	(π)	Ө (= Ne μ)	NA	AR	He	Ho
***G*. *macrorhynchus***		**62**	**32: 30**	**16**	**0.667**	**0.002**		**64**	**62.690**	**0.828**	**0.779**
	Azores Islands	16	14: 2	4	0.517	0.001	0.0022	45	28.200	0.822	0.783
	Canary Islands	26	14: 12	11	0.697	0.002	0.0101	50	27.299	0.806	0.804
	French Polynesia	20	4: 16	3	0.582	0.001	0.0007	44	26.003	0.776	0.782
***G*. *melas***		**57**	**50: 7**	**12**	**0.474**	**0.001**		**45**	**44.268**	**0.644**	**0.616**
	Faeroe Islands	50	48: 2	9	0.263	0.001	0.0029	41	20.389	0.644	0.617
	Iberian Peninsula	7*	2: 5	6	0.928	0.003	0.0044	21	17.680	0.603	0.525

*The only *G*. *macrorhynchus* individual from the Iberian Peninsula was not taken into account for these calculations.

N: sample size; M: Male; F: Female; Nh, Number of haplotypes; Hd, Haplotype diversity; (π), Nucleotide diversity; Ө (= Ne μ), theta is equal to the effective population size (Ne) times the mutation rate of the species per site per generation (μ). NA, Number of alleles per locus. AR, allelic richness. He and Ho, heterozygosity observed and expected respectively

Twenty eight D-Loop haplotypes of 703 base pairs (bp) were found. They are available in GenBank database (https://www.ncbi.nlm.nih.gov/genbank/) under the accession numbers KJ740360-KJ740387. High haplotype diversity and low nucleotide diversity were found in both species but *G*. *melas* had lower haplotype and nucleotide diversities, as well as lower number of haplotypes and lower theta values (Table 1) than *G*. *macrorynchus*.

Two microsatellite loci (EV94MN and 468/469) might exhibit possible null alleles (detected with MICROCHECKER) and were discarded from further analyses. Null alleles and scoring errors were not detected for six microsatellite loci. Linkage disequilibrium was not significant and none of these loci deviated significantly from Hardy-Weinberg equilibrium, thus they were used in further population analysis. The six loci were very variable (S1 Table). Species-specific alleles were present in all loci (except EV37NM and 415/416 for the species *G*. *melas;* S1 Table) and allowed identification of interspecific hybrids. No significant differences between expected and observed heterozygosities were detected, and low F_IS_ values were found (S1 Table).

**Fig 3:**
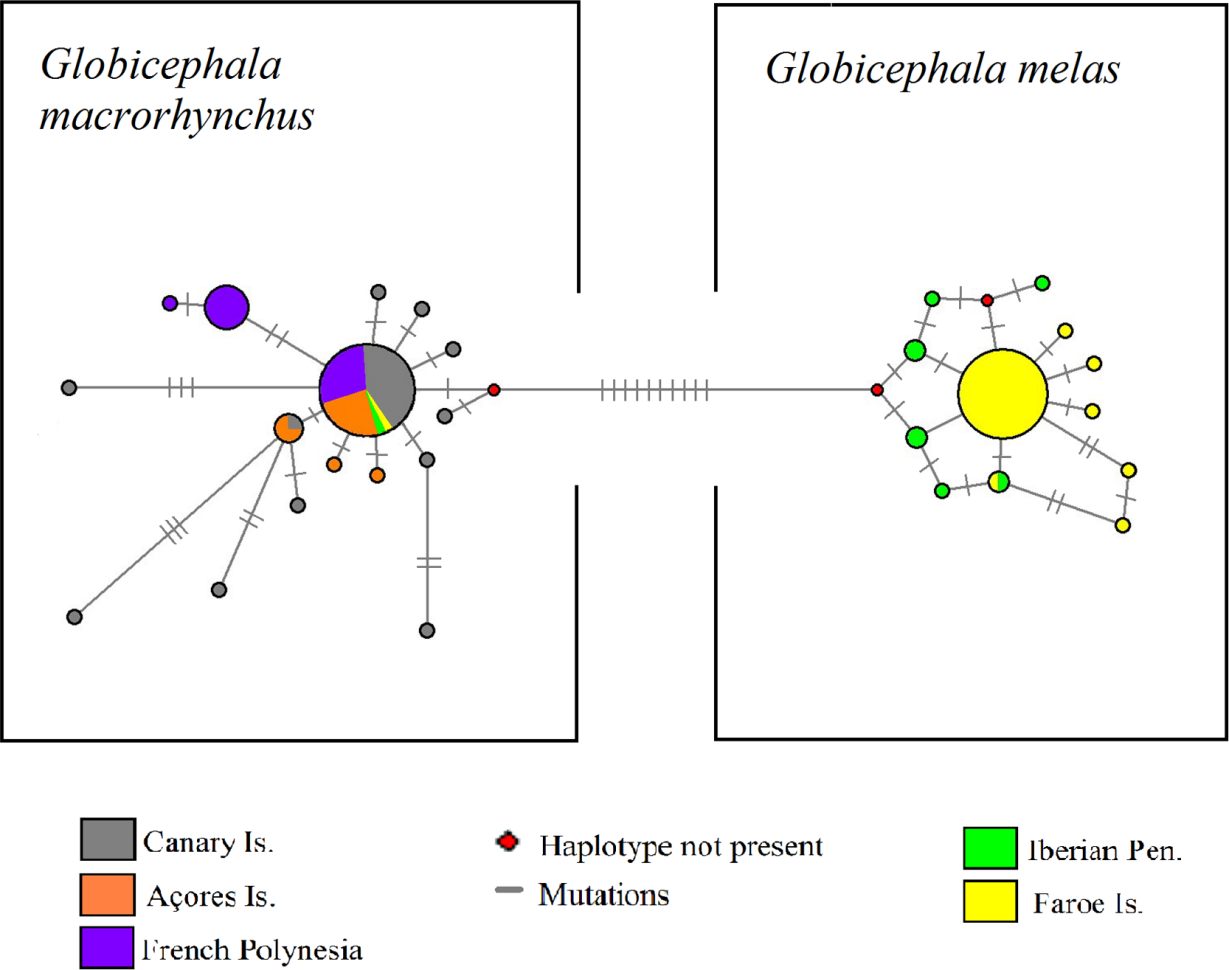
Mitochondrial haplotypes network. Median-Joining network representing the relationships among D-loop mitochondrial haplotypes. Circle sizes are proportional to the frequency of each haplotype. Different locations are represented in different colors. Each species is clustered in a square. Mutations are represented as perpendicular bars in the branches. Inferred haplotypes not present in the dataset are symbolized with a small red circle.

*G*. *melas* and *G*. *macrorhynchus* were unambiguously differentiated in the haplotype network (Fig 3), with 10 different mutations and two inferred haplotypes between them. For the two species the haplotype network exhibited a star-like shape that may indicate recent population expansion [66]. No genetic population differentiation was found in *G*. *melas*, neither in *G*. *macrorhynchus* for Atlantic Ocean samples (S2 Table). Recent bottlenecks were not detected for any *Globicephala* species in this study.

### Interspecific genetic relationships

**Fig 4:**
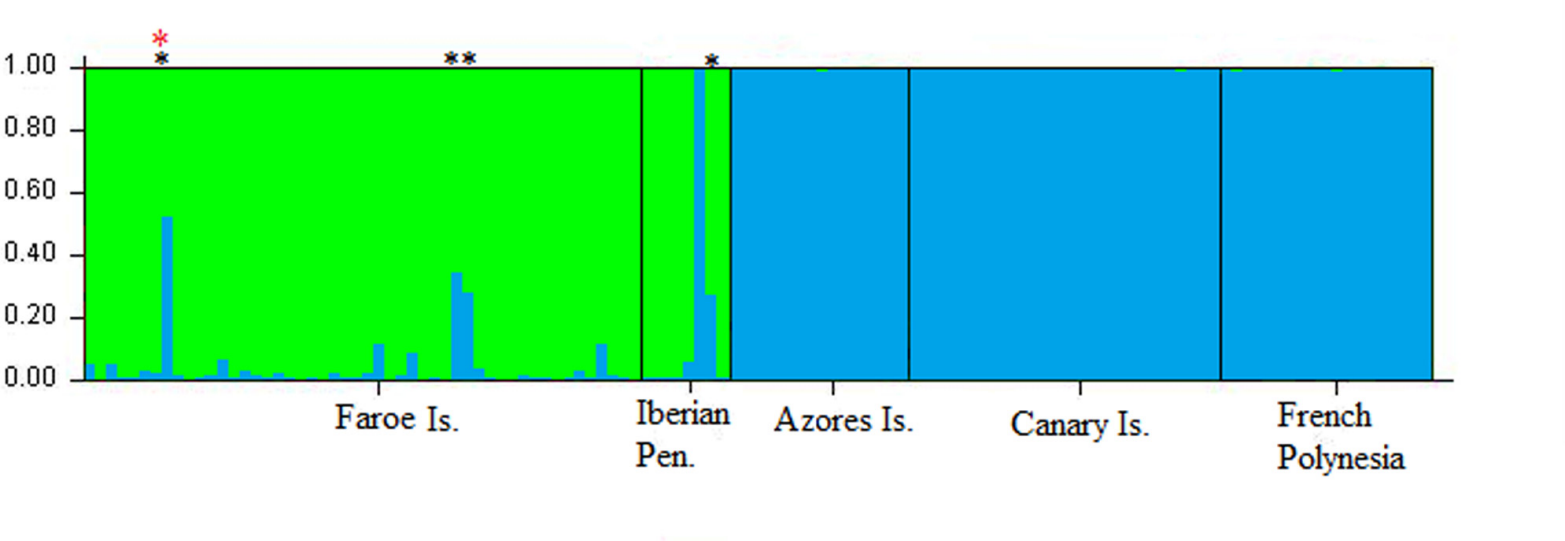
Genetic introgression in *Globicephala* species. Introgression detected with STRUCTURE 2.3.1 software and represented as membership of each *Globicephala* species. Each bar represents one individual and each color means one species: green for *G*. *melas* and blue for *G*. *macrorhynchus*. Hybrids are indicated with a black asterisk and the nuclear-mitochondrial discordant with a red asterisk. Sampling locations are indicated below the graph.

Hybridization and introgression were detected from the analyzed microsatellite loci (Fig 4), which had a high discrimination power in STRUCTURE (99.8%) and NewHybrids (98.4%). Four post-F1 hybrids were identified (Fig 4; Table 2) in *G*. *melas* while none was found in *G*. *macrorhynchus* samples. Three of the four hybrids (Table 2) originated from crosses between *G*. *melas* females and *G*. *macrorhynchus* males, as deduced from their mitochondrial DNA *G*. *melas*-type. The other hybrid was morphologically identified as *G*. *melas*, and genetically assigned to *G*. *melas* from nuclear markers, but had *G*. *macrorhynchus*-type mitochondrial DNA. This evident morphological and nuclear-mitochondrial discordance can be caused by repeated backcrosses of a hybrid issued from a cross [*G*. *macrorhynchus* female x *G*. *melas* male] to *G*. *melas*. These findings demonstrate that introgression is asymmetrical, with the genome of *G*. *macrorhynchus* entering into *G*. *melas* genome and not in the opposite direction. In the studied population, hybrids represented 7.02% of *G*. *melas* individuals with an average of 6.2% introgressed membership estimated from STRUCTURE (Fig 4; Table 2). The number of alleles in *G*. *melas* was indeed higher when hybrids were included in the dataset (45 alleles; Table 1) than when they were not (39 alleles; S1 Table).

**Table 2:** Hybridization in Pilot whales.

	New Hybrids 1.0			STRUCTURE 2.3.1	
	Hybrid	Pure GME	Pure GMA	Memb.GME	Memb.GMA
*G*. *macrorhynchus*	0	0.000	0.985	0.003 (0.002–0.004)	0.996 (0.995–0.997)
*G*. *melas*	4	0.966	0.000	0.937 (0.902–0.972)	0.062 (0.027–0.097)
All 4 hybrids	4	0.665	0.000	0.629 (0.518–0.740)	0.371 (0.260–0.482)
Hybrid F 07	F2 GME	0.524	0.000	0.465 (0.354–0.576)	0.535 (0.424–0.646)
Hybrid F 12	Bx GME	0.711	0.000	0.650 (0.539–0.761)			0.350 (0.239–0.461)
Hybrid F 33	Bx GME	0.671	0.000	0.742 (0.631–0.853)	0.258 (0.147–0.369)
Hybrid IP 53	F2 GME	0.752	0.000	0.674 (0.563–0.785)	0.326 (0.215–0.437)

Hybridization scores in proportions per species, per introgressed individuals and for each descendent of hybrid calculated with two different methods (NewHybrids 1.0 and STRUCTURE 2.3.1 software) STRUCTURE confidence intervals (95%) are included in brackets. GME: *Globicephala melas*; GMA: *Globicephala macrorhynchus*; Memb.: Membership; F: Feroe Islands; IP: Iberian Peninsula; F2: second generation; Bx: Backcrossing.

A high interspecific genetic interchange between species, measured as effective immigrants, was obtained from two different statistical approaches. Based on private alleles and microsatellite data, the interchange between species was estimated at 1.131 individuals per generation. From a coalescence method and mitochondrial sequences, interspecific gene flow was estimated at 0.429 individuals per generation. It increased to 1.463 individuals per generation when employing microsatellite data with *G*. *melas* being always the receptor species.

### Coalescent evolutionary and demographic inferences

**Fig 5:**
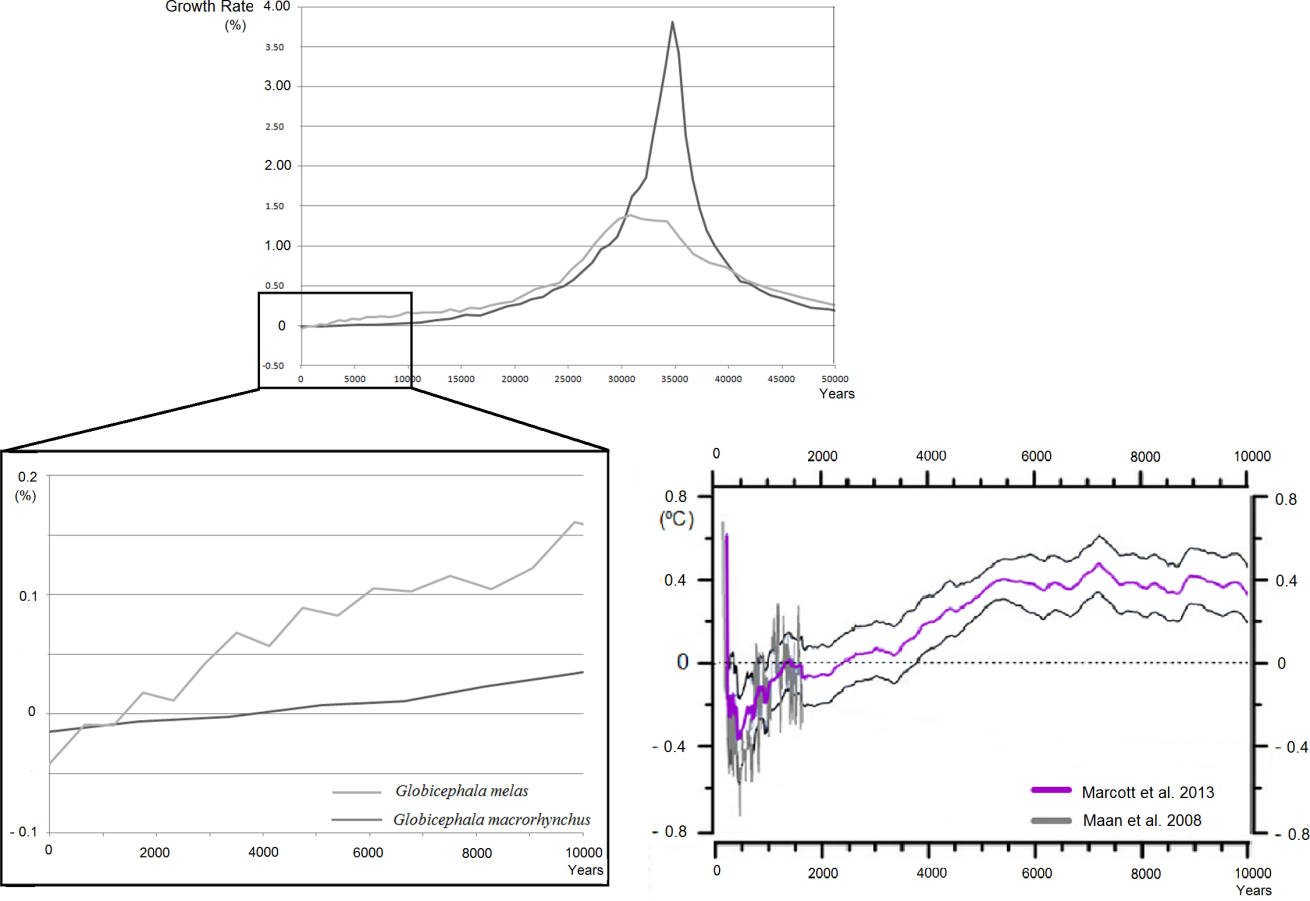
Pilot whale historical growth rates. Reconstruction of the historical growth rates of both pilot whale species. The graph at the left side of the bottom shows the growth rates during the last 10000 years. *G*. *macrorhynchus* is represented in dark grey and *G*. *melas* is in light grey. The graph at the right side of the bottom represents the global temperature anomalies over the past 10000 years compared to historic average (1961–1990), this image is adapted from Marcott et al. [36]

The divergence time between pilot whale species was estimated to occur about 648 500 years ago (standard deviation = 4817.4 years; 95% Highest Posterior Density = 363 600–961 900). A reconstruction of historical population growth of both species revealed a very pronounced peak in *G*. *macrorhynchus* growth rate between 30 000–40 000 years ago. After this point, the growth rate of *G*. *macrorhynchus* decreased rapidly, remaining below that of *G*. *melas*. In the last 10 000 years, the situation changed for *G*. *melas* that suffered a drastic decrease in growth rates and reaching values lower than those of *G*. *macrorhynchus* (Fig 5). Historical growth rate of *G*. *melas* in this later period seems to match historical temperature anomalies (Fig 5) during the Holocene (starting 11000 years ago), following the last glacial period in late Pleistocene (30000–25000 years ago). Significant polynomial regressions were found between Holocene paleotemperatures and historical growth rate of the two species, stronger for *G*. *melas* (R^2^ = 0.9187) than for G. *macrorhynchus* (R^2^ = 0.7799).

## Discussion

This is the first report of genetic admixture and interactions between pilot whale species promoted by distribution shifts that might be caused by global temperature warming., These results may be extended to other cetacean species, since shifts in distribution ranges resulting from climate warming are expected to occur in many cetaceans e.g. [16, 17, 67]. However, since the results of this study are based on correlational inferences and the period considered is relatively long, an alternative scenario of continuous limited admixture between species regardless of climate changes may also be possible.

Previous studies have reported the strong relationship between sea surface temperature (SST) and pilot whale distribution, migration and abundance [16, 18–24, 26–28, 68]. Water temperature increased significantly in the last decades (Figs 1 and 2). Along with temperature, the distribution limit of *Globicephala macrorhynchus* in the Northeast Atlantic shifted 3° latitude in only two decades. In 1988 it was described in the western edge of the Cantabrian Sea within Spanish waters at 43°36’ N [69], while in 2010 it occurs in Charente-Maritime in France at 46°06’ N [70]. Moreover, strandings of this species (confirmed by genetic analyses) have been recently reported in this area of France providing another unequivocal signal of the northward displacement of this species [71, 72]. Climate change could therefore facilitate mating between *G*. *macrorhynchus* and *G*. *melas* by widening the area of co-occurrence of the two species, especially during the reproductive peaks in summer (Fig 2B). In the North Atlantic Ocean, reproduction happens during the warmer months and the observation of larger groups coincides with a higher proportion of mixed pods [73–74]. The warmer SST anomalies in the last decades in northern latitudes (Fig 2) are particularly intense and coincide with the reproduction season of *Globicephala* (Fig 2B). The increase in water temperature during mating season (Fig 2B) facilitates northward incursions of *G*. *macrorhynchus*, and therefore may have increased opportunities for interspecific mating between pilot whales.

A process of introgressive hybridization is happening in Northeast Atlantic *G*. *melas*. Nuclear genetic markers revealed only unidirectional introgression of *G*. *macrorhynchus* DNA into *G*. *melas* genome. Between-species gene flow estimates were higher for microsatellites (1.14–1.46 individuals per generation) than for mitochondrial DNA (0.43 individuals per generation), as it was also reported for intraspecific gene flow in pilot whales [75]. The high level of introgression found in this study, quite uncommon for mammal species, might be explained by a recent species divergence. The divergence time between the two pilot whales was estimated about 648500 (± 5000) years ago. This is a very recent split from the last common ancestor and would explain the relative permeability of interspecific reproductive barriers [76–78].

Hybridization is more frequent in areas where population density is low and where species are near the edge of their ecological range [79]. This could be the case of *Globicephala* in their northernmost distribution area. Lower population variation suggests reduced population size of *G*. *melas* in comparison with *G*. *macrorhynchus*. Differential selection and other possible factors may explain this difference between species [80, 81]. Bottlenecks and genetic or cultural hitchhiking also leave similar footprints in DNA [15, 82]. Although we did not detect any recent bottleneck event in *G*. *melas* from our analysis, matrilineal social structure together with high mortality can reduce mitochondrial DNA diversity [81]. This could explain reduced diversity in *G*. *melas* since this species has been and continues to be hunted. Climate warming has accelerated in recent decades, and it is possible that the population reduction is too recent for detecting significant genetic evidence of bottlenecks. Another possible explanation would be a sustained population decline over a long period, which is not viewed as a bottleneck. Our reconstruction of historical population growth rates suggests that North Atlantic *G*. *melas* has been declining since the last glacial period in the late Pleistocene (30000–25000 years ago), and more intensely during the Holocene when population growth became negative (Fig 5). The intense warming that occurred throughout the Holocene (e.g. [83]) could have contributed to *G*. *melas* decline, as suggested by the correlation between temperature anomalies and the historical population growth rate of this species.

As a final remark, historical and present population reductions, global warming, climate change and other possible factors, like human-mediated stressors such as marine traffic or cultural hunts, seem to negatively affect *G*. *melas*. An uncertain future is waiting for this species under climate change and global warming scenarios. *G*. *melas* could disappear from the Northeast Atlantic Ocean, as it has already disappeared from the North Pacific, about 800–1200 years ago in Japan [84] and 3500–2500 years ago in Alaska [85]. To preserve *G*. *melas* in northern latitudes it seems advisable to develop a monitoring program and implement a conservation plan for this species.

## Supporting Information

S1 Table Microsatellite variation for each species (without suspected hybrids). NA: Number of Alleles; AR: Allele Range; EA: Exclusive Alleles; He: Expected heterozygosity; Ho: Observed heterozygosity; FIS: Inbreeding coefficient; HWE: p-value of Hardy-Weinberg equilibrium (no-significant values after Bonferroni correction).
(DOCX)

S2 Table Microsatellite and mtDNA population genetic distances (F_ST_) without suspected hybrids. Pairwise F_ST_ estimates based on mtDNA (below diagonal) and microsatellite loci (above diagonal). Significant P values are in bold. GME: *Globicephala melas*; GMA: *G*. *macrorhynchus*.
(DOCX)
