# Supplementary material for: Interspecific Hybridization in Pilot Whales and Asymmetric Genetic Introgression in Northern Globicephala melas under the Scenario of Global Warming
Source: PLoS One. 2016 Aug 10;11(8):e0160080. doi: 10.1371/journal.pone.0160080 (PMC4980017; doi:10.1371/journal.pone.0160080)
Supplement: S1 Table — NA: Number of Alleles; AR: Allele Range; EA: Exclusive Alleles; He: Expected heterozygosity; Ho: Observed heterozygosity; FIS: Inbreeding coefficient; HWE: p-value of Hardy-Weinberg equilibrium (no-significant values after Bonferroni correction). (DOCX) [file pone.0160080.s001.docx]

S1Table: **Microsatellite variation for each species (without suspected hybrids)**

|  |  | ***G. melas*** | ***G.macrorhynchus*** |
| --- | --- | --- | --- |
| EV37NM | NA | 7 | 10 |
|  | AR | 184-198 | 164-200 |
|  | EA | - | 3 |
|  | He | 0.772 | 0.797 |
|  | Ho | 0.755 | 0.661 |
|  | FIS | 0.040 | 0.146 |
|  | HWE | 0.764 | 0.078 |
| 199/200 | NA | 3 | 10 |
|  | AR | 110-132 | 120-142 |
|  | EA | 2 | 9 |
|  | He | 0.314 | 0.862 |
|  | Ho | 0.304 | 0.797 |
|  | FIS | 0.110 | 0.098 |
|  | HWE | 0.064 | 0.114 |
| 415/416 | NA | 5 | 11 |
|  | AR | 230-238 | 212-242 |
|  | EA | - | 6 |
|  | He | 0.637 | 0.854 |
|  | Ho | 0.597 | 0.853 |
|  | FIS | 0.088 | 0.030 |
|  | HWE | 0.763 | 0.120 |
| 417/418 | NA | 3 | 7 |
|  | AR | 183-189 | 173-187 |
|  | EA | 1 | 4 |
|  | He | 0.515 | 0.704 |
|  | Ho | 0.511 | 0.698 |
|  | FIS | 0.031 | 0.038 |
|  | HWE | 0.999 | 0.370 |
| 409/470 | NA | 11 | 13 |
|  | AR | 176-200 | 174-204 |
|  | EA | 2 | 4 |
|  | He | 0.774 | 0.888 |
|  | Ho | 0.704 | 0.878 |
|  | FIS | 0.062 | 0.058 |
|  | HWE | 0.226 | 0.042 |
| 464/465 | NA | 10 | 12 |
|  | AR | 138-158 | 118-152 |
|  | EA | 2 | 4 |
|  | He | 0.780 | 0.889 |
|  | Ho | 0.745 | 0.870 |
|  | FIS | 0.055 | 0.035 |
|  | HWE | 0.073 | 0.399 |
| All loci | NA | 39 | 63 |
|  | AR | 110-238 | 118-242 |
|  | EA | 7 | 30 |
|  | He | 0.624 | 0.832 |
|  | Ho | 0.595 | 0.792 |
|  | FIS | 0.053 | 0.068 |

NA: Number of Alleles; AR: Allele Range; EA: Exclusive Alleles; He: Expected heterozygosity; Ho: Observed heterozygosity; FIS: Inbreeding coefficient; HWE: p-value of Hardy-Weinberg equilibrium (no-significant values after Bonferroni correction).
