# Supplementary material for: Interspecific Hybridization in Pilot Whales and Asymmetric Genetic Introgression in Northern Globicephala melas under the Scenario of Global Warming
Source: PLoS One. 2016 Aug 10;11(8):e0160080. doi: 10.1371/journal.pone.0160080 (PMC4980017; doi:10.1371/journal.pone.0160080)
Supplement: S2 Table — Pairwise FST estimates based on mtDNA (below diagonal) and microsatellite loci (above diagonal). Significant P values are in bold. GME: Globicephala melas; GMA: G. macrorhynchus. (DOCX) [file pone.0160080.s002.docx]

S2 Table: **Microsatellite and mtDNA population genetic distances (F_ST_) without suspected hybrids.**

| Localities | GME_Faeroe I. | GME_Iberian P. | GMA_Canary I. | GMA_Azores I. | GMA_ F. Polynesia |
| --- | --- | --- | --- | --- | --- |
| GME_Faeroe I. | - | **-0.0239** | 0.1601 | 0.1829 | 0.1160 |
| GME_Iberian P. | **0.0739** | - | 0.1713 | 0.1957 | 0.1425 |
| GMA_Canary I. | 0.9627 | 0.9284 | - | **0.0088** | 0.1028 |
| GMA_Azores I. | 0.9666 | 0.9273 | **-0.0221** | - | 0.0937 |
| GMA_ F. Polynesia | 0.9513 | 0.8900 | 0.3954 | 0.4017 | - |

Pairwise F_ST_ estimates based on mtDNA (below diagonal) and microsatellite loci (above diagonal). Significant P values are in bold. GME: *Globicephala melas*; GMA: *G. macrorhynchus*.
